# Chlorhexidine Substantivity on Salivary Flora and Plaque-Like Biofilm: An *In Situ* Model

**DOI:** 10.1371/journal.pone.0083522

**Published:** 2013-12-27

**Authors:** Lucía García-Caballero, Victor Quintas, Isabel Prada-López, Juan Seoane, Nikos Donos, Inmaculada Tomás

**Affiliations:** 1 Oral Sciences Research Group, School of Medicine and Dentistry, Santiago de Compostela University, Santiago de Compostela, Spain; 2 Periodontology Unit, UCL Eastman Dental Institute, London, United Kingdom; University of Florida, College of Dentistry & The Emerging Pathogens Institute, United States of America

## Abstract

**Objective:**

To evaluate the *in situ* antibacterial activity of a mouthrinse with 0.2% Chlorhexidine (M-0.2% CHX) on undisturbed *de novo* plaque-like biofilm (PL-biofilm) and on salivary flora up to 7 hours after its application.

**Methods:**

A special acrylic appliance was designed, with 3 inserted glass disks on each buccal side, allowing for PL-biofilm growth. Fifteen healthy volunteers wore the appliance for 48 hours and then performed an M-0.2% CHX; disks were removed at 30 seconds and 1, 3, 5 and 7 hours after the mouth-rinsing. Applying a washout period, saliva samples were collected from each volunteer at 30 seconds and 1, 3, 5 and 7 hours after performing an M-0.2% CHX. The PL-biofilm and saliva samples were analysed by confocal laser scanning and epifluorescence microscopes, respectively.

**Results:**

At 30 seconds after M-0.2% CHX, the levels of viable bacteria detected in saliva were significantly lower than those observed in PL-biofilm. The difference in the percentage of live bacteria detected in saliva was significantly higher than that observed in PL-biofilm at 5 and 7 hours after M-0.2% CHX.

**Conclusion:**

After a single mouthrinse of the 0.2% CHX formulation tested in the present study, the 2-day PL-biofilm presented a significantly higher resistance to this antiseptic *in situ* than that observed in salivary flora. However, this 0.2% CHX formulation showed a higher substantivity on PL-biofilm than on salivary flora at 5 and 7 hours after mouth-rinsing, which could be related to the slower growth rate of PL-biofilm and the possible reservoir function for antimicrobial agents associated with the undisturbed *de novo* PL-biofilm.

## Introduction

The *in vitro* development of biofilm models have led to significant advances in the study of oral biofilms [1]. However, *in vitro* oral biofilm models tend to involve limited numbers of species and, in addition, they are created under conditions that still cannot adequately reflect the physiological situation in the oral cavity [2]–[4]. Factors related to the oral cavity, such as the turnover rate of saliva, the ability of antibacterial substances to adhere to the pellicle of the tooth or the surface of soft tissues in order to achieve their effects, and the interaction with unculturable bacteria, cannot be modelled in *in vitro* experiments [5]. Consequently, at the present time, the scientific community recognizes that *in vitro* models cannot guarantee the creation of oral biofilms whose composition and structure is comparable with those that form *in situ* [2]–[4], [6]. For this reason, there is a need to develop *in situ* biofilm models that can subsequently be analysed intact *ex vivo* [2], [7], [8].

Studies have been published in the literature in which the *in situ* antimicrobial activity of CHX on the plaque-like biofilm (PL-biofilm) has been evaluated using microbiological plate culture techniques [9], [10]. However, numerous disadvantages associated with the use of culture-dependent methods are well known [5], [11]. Since Netuschil first used fluorescence techniques to investigate dental plaque in 1983 [12], numerous authors have used fluorescence methods to study the *in situ* antibacterial effect of CHX on PL-biofilm. A common methodological characteristic of all of these studies is that evaluation of the supragingival bacterial plaque was performed on material previously removed from the surface of the tooth [13]–[15], whereas the subgingival bacterial plaque was obtained by paper point sampling or by mechanical debridement [9], [16]; this is likely to disturb the delicate three-dimensional relationship of the cells, matrix, space, and substrate [17]–[19]. Another disadvantage of this method, in which the dental plaque is disturbed, is that the level of penetration of an antimicrobial agent into the PL-biofilm cannot be evaluated as the samples are dispersed for analysis [14]. This methodology therefore provides an inadequate study of the architecture and organization of *in vivo* PL-biofilm, as well as of the action of antimicrobial agents on its structure [4], [20].

As a result, and in order to improve the methodology of such studies, special removable appliances that include a number of disks on which growth of the PL-biofilm can take place have been designed [3], [20]–[22]. Subsequently, this undisturbed PL-biofilm is analysed using confocal laser scanning microscopy (CLSM) and fluorescence solutions that permit the simultaneous study of the three-dimensional structure of the biofilm and the evaluation of bacterial viability [3], [20]–[22]. Other techniques such as fluorescence-labelled antibodies and fluorescence hybridisation (FISH) have been frequently used in combination with CLSM to analyse bacterial topography of *in situ* undisturbed PL-biofilm [18], [19], [23], [24]. With CLSM, biofilms can be studied in their natural hydrated state, with no requirement for dehydration, fixation, or staining [2], [20], [25]. In addition, the optical sectioning properties of CLSM mean that very thin optical sections in the horizontal plane (X–Y axes) can be taken at 0.5 to 2 µm intervals, at increasing depths through the biofilm (from the surface of the biofilm to its base), that are free from out-of-focus blurring [5], [18], [25], [26].

Consequently, at present, the scientific community considers that the methodological design based on using of special removable appliances (including disks) to obtain biofilm samples and its analysis by CLSM (in combination with other microscopic and microbiological techniques) is the most suitable approach for studying the *in situ* architecture and physiology of undisturbed PL-biofilm formed on surfaces, as well as the antibacterial effect of antimicrobials on this microbial structure [8], [17], [20]. However, there are few studies in the literature in which the effects of CHX on *in situ* undisturbed PL-biofilm have been investigated applying CLSM together with bacterial viability techniques [3], [25], [27], [28].

The aim of the present study was to evaluate the *in situ* antibacterial activity of a 0.2% CHX mouthrinse on undisturbed *de novo* PL-biofilm up to 7 hours after its application, comparing the results with those obtained on salivary flora.

## Materials and Methods

This was a randomised, double-blind, crossover study of the antibacterial efficacy of CHX on an *in situ* model of PL-biofilm growth.

### Selection of the study group

The study group was formed of 15 systemically healthy adult volunteers between 20 and 45 years of age and who presented a good oral health status: a minimum of 24 permanent teeth with no evidence of gingivitis or periodontitis (Community Periodontal Index score  = 0) [29], and an absence of untreated caries. The following exclusion criteria were applied: smoker, presence of dental prostheses or orthodontic devices, antibiotic treatment or routine use of oral antiseptics during the previous 3 months, and presence of any systemic disease that could alter the production or composition of the saliva. A professional tooth cleaning was performed on all volunteers before starting the study.

This project was approved (number 2012/394) by the Clinical Research Ethics Committee of Galicia. Written informed consent was obtained from all participants in the study.

### Production of the disk-holding splint

**Figure 1:**
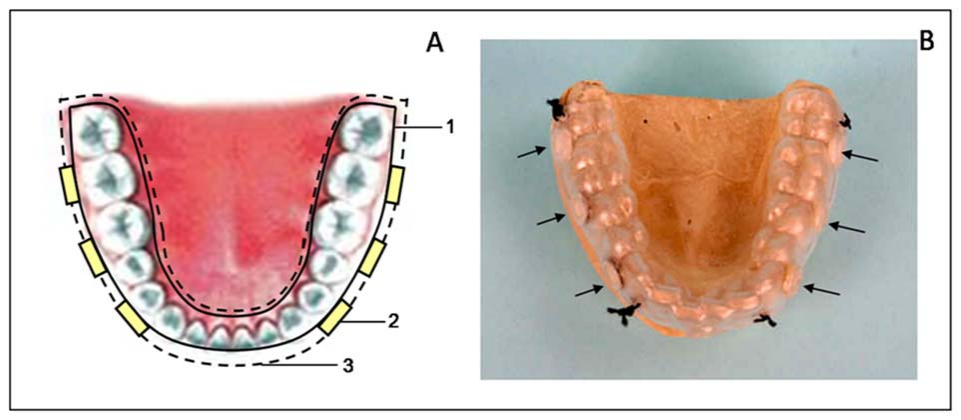
Individualized splint of the lower arch. A) Parts of the individualized splint: 1. internal vinyl sheet; 2. polished glass discs; 3. fenestrated external vinyl sheet. B) Clinical view of the individualized splint with the glass discs inserted (arrows).

After considering a number of previously described *in situ* models [3], [20]–[22], an individualised splint of the lower arch was created for each volunteer, which was able to hold 6 glass disks (6 mm diameter, 1 mm thickness) polished at 800 grit. The characteristics of this splint have been previously described by authors [30]. This splint was formed of 2 vinyl sheets, an internal sheet with a thickness of 1 mm to which 6 discs were attached, and an external sheet with a thickness of 0.5 mm that was fenestrated (patent number ES2380252B2; Figure 1).

The splint with the glass disks was worn by the volunteer for 48 hours to favour growth of the PL-biofilm, withdrawing it from the oral cavity only during meals (it was stored in a physiological sterile saline solution) and to perform oral hygiene using only the mechanical removal of bacterial plaque with water without the use of toothpaste or mouthrinse.

### Application of the Chlorhexidine protocol on PL-biofilm (Application 1)

After 48 hours, the glass disks were withdrawn one on one from the splint from each volunteer (from right to left; in a distal-mesial direction) at baseline, 30 seconds, 1, 3, 5 and 7 hours after performing the following mouthrinses under supervision:

-A single, 30-second mouthrinse with 10 mL of sterile water (negative control) (M-water).

OR

-A single, 30-second mouthrinse with 10 mL of 0.2% CHX (Oraldine Perio®, Johnson and Johnson, Madrid, Spain) (M-0.2% CHX).

On the day of the experiment, the volunteers were not allowed to eat or drink during the course of the tests.

Collection of the different PL-biofilm samples started at 11:50 AM (baseline sample) and finished at 7 PM (final sample obtained 7 hours after performing the mouthrinse).

Using a balanced randomisation system, all volunteers performed the 2 mouthrinses, with a washout period of 2 weeks between each test.

### Application of the Chlorhexidine protocol on salivary flora (Application 2)

Applying a washout period (2 weeks) from application of the CHX protocol on PL-biofilm, unstimulated saliva samples (1 ml) were collected from each volunteer (in absence of the disk-holding splint) at 30 seconds and 1, 3, 5 and 7 hours after performing the following mouthrinses under supervision:

-A single, 30-second mouthrinse with 10 mL of sterile water (negative control) (M-water).

OR

-A single, 30-second mouthrinse with 10 mL of 0.2% CHX (Oraldine Perio®, Johnson and Johnson, Madrid, Spain) (M-0.2% CHX).

On the day of the experiment, the volunteers were not allowed to eat or drink during the course of the tests.

Collection of the different PL-biofilm samples started at 11:50 AM (baseline sample) and finished at 7 PM (final sample obtained 7 hours after performing the mouthrinse).

Using a balanced randomisation system, all volunteers performed 2 mouthrinses, with a washout period of 2 weeks between each test. The unstimulated saliva samples were collected using a previously described method (the spitting method) [31].

### Processing of the samples of PL-biofilm

The characteristics of the LIVE/DEAD® BacLight^TM^ fluorescence solution (Molecular Probes, Leiden, The Netherlands), as well as its preparation, have been described by authors in a previous study [11].

The glass disks were withdrawn from the splint and were immediately submerged in 100 µl of fluorescence solution and were kept in darkness at room temperature for 15 minutes. Microscope observation was performed by a single investigator who was unaware of the study design, using a Leica TCS SP2 laser scanning spectral confocal microscope (Leica Microsystems Heidelberg GmbH, Mannheim, Germany) with an HCX APOL 63×/0.9 water-immersion lens.

Four randomly selected fields or XYZ series in the central part of each disk were evaluated. These fields were considered as representative of the whole after general examination. Fluorescence emission was determined in a series of XY images in which each image corresponded with each one of the Z positions (depth). The optical sections were scanned in one micron sections from the surface of the biofilm to its base, measuring the maximum thickness of the field and subsequently the mean thickness of the biofilm of the corresponding sample. In accordance with other authors [32], the maximum thickness of biofilm field was defined as the distance between the substrate and the peaks of the highest cell clusters. The biofilm maximum thickness of each field was divided into 3 zones or equivalent layers: outer layer (layer 1), middle layer (layer 2) and inner layer (layer 3).

The capture of the data was done with the same settings in all cases. The spatial scan mode (xyz) and the 1024×1024 pixels scan format resolution were used. The Argon-ion and DPSS laser were used at a 13% and 78% of maximum intensity, respectively. The values for the pinhole, zoom and scan speed were 121.58 microns, 1 and 400 Hz, respectively. The only values that were different depending on the sample were the offset (range between −1% to 1%) and PMT gain which was different for channel red and green been higher for red in basal samples and higher for green in 30 seconds and the following in time samples. These values were always adjusted to get a good quality capture without background noise, avoiding excessive saturation of the brightest pixels of the image. As the technician was blind to the experiment, they were advised to make the adjustments always consistent with what was seeing by the objective of the microscope, obtaining an image which was the closest as possible to reality.

Quantification of bacterial viability in the series of XY images was determined using cytofluorographic analysis (Leica Confocal Software). In this analysis, the images of each fluorochrome were defined as “channels” (SYTO 9 occupies the green channel and PI the red channel). Square capture masks were used to measure the area occupied (µm^2^) by the pixels in each channel, determining the total area occupied by the biofilm and the corresponding percentage of viability. The intensity ranges that were considered as positive signal were between 100 and 255. Determination of the mean percentage of bacterial viability in each field required sections with a minimum area of biofilm of 250 µm^2^, and the mean percentage of bacterial viability of the biofilm was calculated for the corresponding sample and for each biofilm layer.

### Processing of the saliva samples

The characteristics of the LIVE/DEAD® BacLight^TM^ fluorescence solution (Molecular Probes, Leiden, The Netherlands), as well as its preparation, have been described by authors in a previous study [11].

Processing of the saliva samples, as well as counting of viable and non-viable bacteria have been described by authors in previous studies [11], [33], [34]. The observations were performed by 2 researchers who were not aware of the study design, using an Olympus BX51 microscope (Olympus, Tokyo, Japan) fitted with a filter set for fluorescein and Texas Red. The excitation/emission maxima are about 480/500 nm for SYTO 9 stain and 490/635 nm for propidium iodide. In relation to objective lens properties, Mag 100× and NA 1.25.

The count of viable and non-viable bacteria was performed at high magnification (×100) on 20 fields (10 fields per slide) that presented a minimum of 100 bacteria (bacterial aggregates were excluded). The mean percentage of viable bacteria was calculated for each saliva sample.

### Statistical analysis

The results were analysed using the PASW® Statistics Base 18 package for Windows (IBM, Madrid, Spain). The intraclass correlation coefficient (ICC, 2-factor model, random effects) and the degree of homogeneity of the elements from the “absolute agreement” perspective were calculated for the intra-observer and inter-observer analysis of the epifluorescence microscopy technique. The data on thickness and bacterial viability in PL-biofilms, as well as bacterial viability in saliva were expressed as mean and standard deviation of the mean. All of the variables analysed presented a normal distribution, which was determined using the Kolmogorov-Smirnov test. One-way ANOVA with repeated measures was used for intra-mouthrinse comparisons using all of the PL-biofilm samples. Two-way ANOVA with repeated measures was used for intra-mouthrinse (differentiating between the 3 biofilm layers), inter-mouthrinse and inter-ecosystem comparisons using all PL-biofilm and saliva samples. Three-way ANOVA with repeated measures was used for inter-mouthrinse (differentiating between the 3 biofilm layers) comparisons using all of the PL-biofilm samples. Pairwise comparisons (with Bonferroni adjustment) were used for the analysis of intra- and inter-mouthrinse (including differentiating between the 3 biofilm layers), as well as inter-ecosystem comparisons between 2 biofilm and saliva samples. Statistical significance was taken as a *P* value less than 0.05.

## Results

In the intra-observer analysis of the epifluorescence microscopy technique, the ICC mean value was 0.92 (*P*<0.001) and in the inter-observer analysis, the ICC mean value was 0.87 (*P*<0.001).

### Thickness, bacterial viability and structural characteristics of PL-biofilm

The mean values of PL-biofilm thickness under basal conditions were 18.15±1.17 µm in the M-water and 22.54±7.64 µm in the M-0.2% CHX (ranged from 11.75 µm to 33.00 µm). The mean values of PL-biofilm viability under basal conditions were 77.33±10.59% in the M-water and 77.89±9.10% in the M-0.2% CHX (ranged from 62.04% to 94.71%).

In relation to the biofilm structural characteristics, an open and heterogeneous architecture model was observed in the biofilm samples, with the presence of fluid-filled channels and bubble-like structures.

### 0.2% CHX: substantivity and influence on PL-biofilm thickness

**Figure 2:**
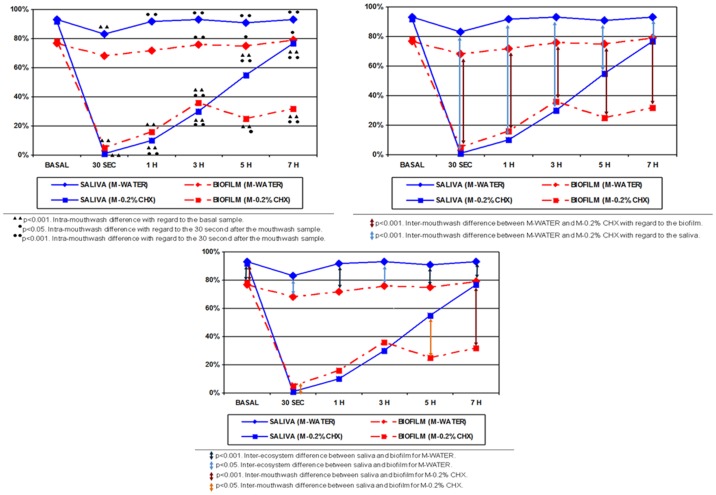
Mean percentages of bacterial viability in saliva and *de novo* PL-biofilm under basal conditions and at different times after a single mouthrinse of sterile water and 0.2% Chlorhexidine. A) Intra-mouthrinse differences; B) Inter-mouthrinse differences; C) Inter-ecosystem differences.

Figure 2 shows the mean percentages of bacterial viability in PL-biofilm and saliva under basal conditions and at 30 seconds and 1, 3, 5, and 7 hours after the M-water and M-0.2% CHX, including the intra-mouthrinse and inter-mouthrinse comparisons.

In comparison with the baseline, the amount of viable bacteria was similar in all of the PL-biofilm samples taken after the M-water. In comparison with the baseline, the percentage of viable bacteria decreased significantly at 30 seconds after the M-0.2% CHX (77.90±9.10% *vs* 5.08±5.82%; p<0.001), detecting a significant antibacterial effect up to 7 hours after the mouthrinse (77.90±9.10% *vs* 31.92±20.17% p<0.001). In comparison with the values obtained 30 seconds after the M-0.2% CHX, a significant recovery of the bacterial population was observed in the later saliva samples (after 3 hours) taken after the mouthrinse. In comparison with M-water, the amount of viable bacteria was significantly lower at 30 seconds after the M-0.2% CHX (68.05±18.99% *vs* 5.08±5.82%, p<0.001), detecting a significant antibacterial effect up to 7 hours after the mouthrinse (79.14±12.42% *vs* 31.92±20.17%; p<0.001).

Table 1 shows the mean percentages of viable bacteria in PL-biofilm under basal conditions and in the samples collected at 30 seconds and 1, 3, 5, and 7 hours after the M-water and M-0.2% CHX, differentiating between the 3 biofilm layers, as well as intra-mouthrinse and inter-mouthrinse comparisons. Differentiating between the 3 biofilm layers, in M-water and M-0.2% CHX, the amount of viable bacteria under basal conditions was significantly higher in the outermost layers with respect to deeper layers. In comparison with M-water, the amount of viable bacteria was significantly lower in the 3 biofilm layers in all of the biofilm samples taken after the M-0.2% CHX (p<0.001 in all comparisons).

**Table 1:** Mean percentages of bacterial viability in PL-biofilm, as well as intra-mouthrinse and inter-mouthrinse comparisons.

Mean ± Standard Deviation (%)						
	BASAL	30 SEC	1 H	3 H	5 H	7 H
**M-water**						
**Layer 1**	85.36±6.55	85.27±13.10	88.30±9.62	90.82±8.91	88.98±8.51	90.93±5.94
**Layer 2**	79.81±7.28	73.08±15.13	78.44±16.56	84.44±10.32	81.34±12.81	85.20±7.06
**Layer 3**	66.83±27.28	45.80±33.35	49.39±29.78	56.73±31.46	55.43±24.91	61.96±24.00
**M-0.2% CHX**						
**Layer 1**	79.94±6.21	5.20±6.19	15.13±15.43	35.42±15.53	21.70±19.74	27.04±22.64
**Layer 2**	82.21±7.83	5.05±6.43	16.54±15.61	36.70±16.42	24.83±20.62	28.65±20.76
**Layer 3**	71.80±17.43	4.97±5.04	15.16±10.21	35.16±14.83	27.44±13.45	40.04±20.37

INTRA-MOUTHRINSE ANALYSIS						
	BASAL	30 SEC	1 H	3 H	5 H	7 H
**M-water**						
**Layer 1 vs. Layer 2**	p<0.05	p≤0.001	p≤0.001	p≤0.001	p≤0.001	p≤0.001
**Layer 1 vs. Layer 3**	–	p≤0.001	p≤0.001	p≤0.001	p≤0.001	p≤0.001
**Layer 2 vs. Layer 3**	–	p≤0.001	p≤0.001	p≤0.001	p≤0.001	p≤0.001
**M-0.2% CHX**						
**Layer 1 vs. Layer 2**	–	–	–	–	–	–
**Layer 1 vs. Layer 3**	–	–	–	–	–	p<0.05
**Layer 2 vs. Layer 3**	p<0.05	–	–	–	–	p<0.05

INTER-MOUTHRINSE ANALYSIS						
	BASAL	30 SEC	1 H	3 H	5 H	7 H
**M-water vs. M-0.2% CHX**						
**Layer 1 vs. Layer 1**	p<0.05	p≤0.001	p≤0.001	p≤0.001	p≤0.001	p≤0.001
**Layer 2 vs. Layer 2**	–	p≤0.001	p≤0.001	p≤0.001	p≤0.001	p≤0.001
**Layer 3 vs. Layer 3**	–	p≤0.001	p≤0.001	p<0.05	p<0.05	p<0.05

Mean percentages of bacterial viability in PL-biofilm under basal conditions and in the samples collected at 30 seconds and 1, 3, 5, and 7 hours after a single mouthrinse of sterile water and 0.2% Chlorhexidine differentiating between the 3 biofilm layers, as well as intra-mouthrinse and inter-mouthrinse comparisons.

– Not a statistically significant difference; M-water  =  a single, 30-second mouthrinse with 10 mL of sterile water; M-0.2% CHX  =  A single, 30-second mouthrinse with 10 mL of 0.2% Chlorhexidine; BASAL = Biofilm sample collected under basal conditions; 30 SEC  =  Biofilm sample collected at 30 seconds after the application of the different mouthrinses; 1 H  =  Biofilm sample collected 1 hour after the application of the different mouthrinses; 3 H  =  Biofilm sample collected 3 hours after the application of the different mouthrinses; 5 H  =  Biofilm sample collected 5 hours after the application of the different mouthrinses; 7 H  =  Biofilm sample collected 7 hours after the application of the different mouthrinses; the biofilm maximum thickness of each field was divided into 3 zones or equivalent layers: outer layer (layer 1), middle layer (layer 2) and inner layer (layer 3).

Table 2 shows the mean values of thickness (µm) in PL-biofilm under basal conditions and at 30 seconds and 1, 3, 5, and 7 hours after the M-water and M-0.2% CHX, as well as the intra-treatment and inter-treatment comparisons. In comparison with the baseline values, M-0.2% CHX provoked a significant reduction effect on biofilm thickness at 1 hour, 3 hours and 7 hours after mouthrinse (p<0.05 in all comparisons). In comparison with M-water, the biofilm thickness was significantly lower in all of the biofilm samples taken after the M-0.2% CHX.

**Table 2:** Mean values of thickness (µm) in PL-biofilm, as well as the intra-treatment and inter-treatment comparisons.

Mean ± Standard Deviation (µm)						
	BASAL	30 SEC	1 H	3 H	5 H	7 H
M-water	19.32±5.41	18.00±2.66	22.25±5.38	20.98±5.12	23.87±4.76	23.90±3.92
M-0.2% CHX	23.43±7.64	15.76±1.87	13.46±2.54	15.35±2.92	17.51±3.88	15.55±2.31

INTRA-MOUTHRINSE ANALYSIS					
	BASAL vs. 30 SEC	BASAL vs. 1H	BASAL vs. 3 H	BASAL vs. 5H	BASAL vs. 7H
		30 SEC vs. 1H	30 SEC vs. 3H	30 SEC vs. 5H	30 SEC vs. 7H
**M-water**	–	–	–	–	–
		p<0.05	–	p<0.05	p<0.05
**M-0.2% CHX**	–	p<0.05	p<0.05	–	p<0.05
		–	–	–	–

INTER-MOUTHRINSE ANALYSIS						
	BASAL	30 SEC	1 H	3 H	5 H	7 H
**M-water vs. M-0.2% CHX**	–	p<0.05	p≤0.001	p≤0.001	p<0.05	p≤0.001

Mean values of thickness (µm) in PL-biofilm under basal conditions and at 30 seconds and 1, 3, 5, and 7 hours after a single mouthrinse of sterile water and 0.2% Chlorhexidine, as well as the intra-treatment and inter-treatment comparisons.

– Not a statistically significant difference; M-water  =  a single, 30-second mouthrinse with 10 mL of sterile water; M-0.2% CHX  =  A single, 30-second mouthrinse with 10 mL of 0.2% Chlorhexidine; BASAL = Biofilm sample collected under basal conditions; 30 SEC  =  Biofilm sample collected at 30 seconds after the application of the different mouthrinses; 1 H  =  Biofilm sample collected 1 hour after the application of the different mouthrinses; 3 H  =  Biofilm sample collected 3 hours after the application of the different mouthrinses; 5 H  =  Biofilm sample collected 5 hours after the application of the different mouthrinses; 7 H  =  Biofilm sample collected 7 hours after the application of the different mouthrinses.

### 0.2% CHX substantivity: PL-biofilm vs. saliva

Figure 2 shows the mean percentages of bacterial viability in PL-biofilm and saliva under basal conditions and at 30 seconds and 1, 3, 5, and 7 hours after the M-water and M-0.2% CHX, including the inter-ecosystem comparisons.

**Figure 3:**
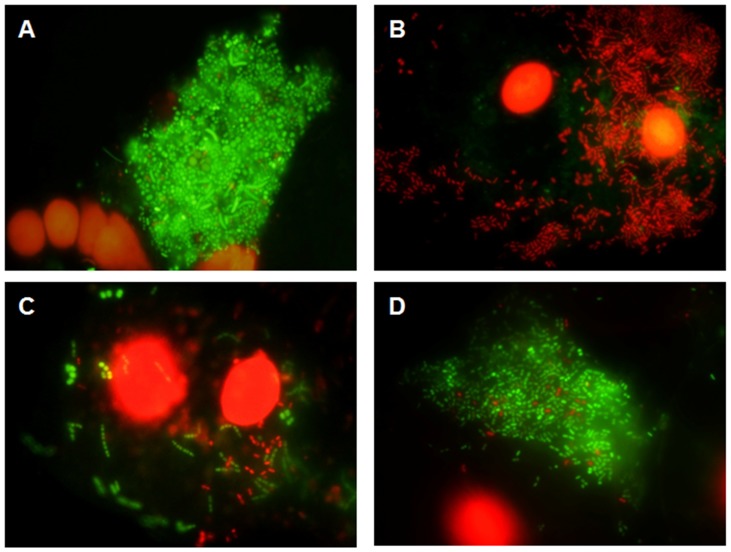
Images representing the changes in bacterial viability in the saliva. A) Sample collected under basal conditions; B), C), D) Sample collected at 30 seconds, 5 and 7 hours respectively after a single mouthrinse of 0.2% Chlorhexidine.

**Figure 4:**
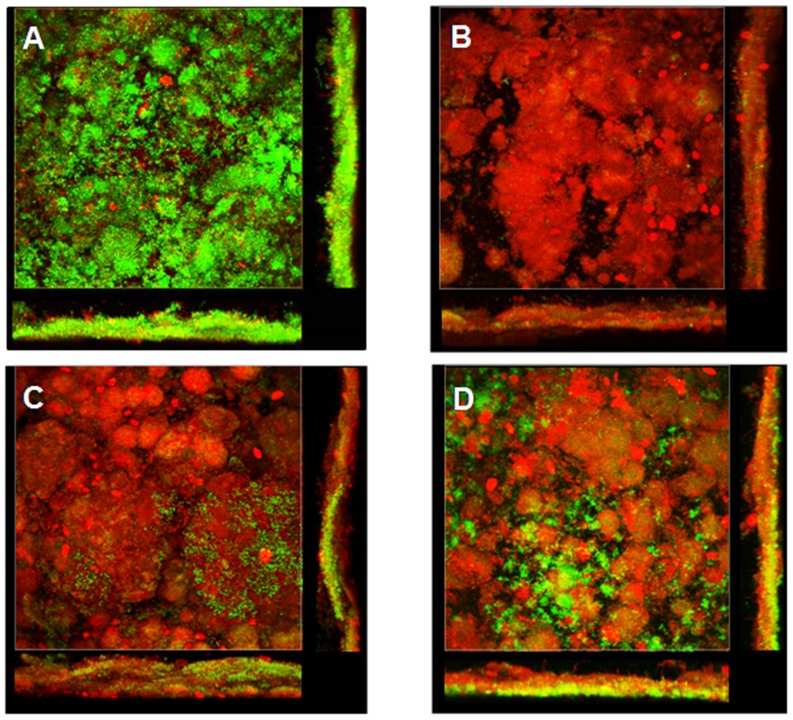
Images representing the changes in bacterial viability in *de novo* PL-biofilm. A) Sample collected under basal conditions; B), C), D) Sample collected at 30 seconds, 3 and 7 hours respectively after a single mouthrinse of 0.2% Chlorhexidine.

The mean bacterial viability in saliva under basal conditions was significantly higher than that detected in PL-biofilm (92.86±1.80% in the M-water and 92.26±4.11% in the M-0.2% CHX *vs* 77.44±7.48% in the M-water and 77.89±9.10% in the M-0.2% CHX; p<0.001). At 30 seconds after M-0.2% CHX, the levels of viable bacteria detected in saliva were significantly lower than those observed in PL-biofilm (0.80±1.20% *vs* 5.08±5.82%; p<0.05). At 1 and 3 hours after M-0.2% CHX, the levels of viable bacteria detected in saliva and PL-biofilm were similar. The difference in the percentage of viable bacteria detected in saliva was significantly higher than that observed in PL-biofilm at 5 hours (55.13±19.96% *vs* 24.66±16.66%; p<0.05) and at 7 hours after M-0.2% CHX (76.86±12.00% *vs* 31.91±20.17%; p<0.001) (Figures 3 and 4).

## Discussion

### Methodological approach

In the majority of published series, measurement of CHX antimicrobial activity in saliva has been performed using plate culture microbiological techniques [35]–[37]. However, in a recent study, we have demonstrated that epifluorescence microscopy using the SYTO 9/propidium iodide dual stain (LIVE/DEAD® BacLight™) was an effective method for quantifying the antibacterial activity of CHX on salivary flora in real-time [11].

In the majority of studies, the number of volunteers who wore the removable appliances ranged from 3 to 10 [18], [20], [26], [38]–[40]. Due to the marked inter-individual variability detected on the characteristics of PL-biofilm [2], [17], [20], [27], [39], a group of 15 individuals was selected in the present series. With regard to the type of removable appliance used to collect the supragingival dental plaque, Wood et al. [17], [41], Watson et al. [4], and Robinson et al. [42] used the “Leeds *in situ* device”, composed of a nylon ring holding an enamel substrate, as it has been previously described [43], on which the PL-biofilm grew. Some authors [24], [39], [44], [45] designed 2 bilateral mandibular stents (spanning the posterior buccal surfaces from the first premolar to first molar), each of which contained several disks, but other different types of individualised acrylic splints for growing PL-biofilm have also been used [2], [3], [5], [20], [22], [25]. In the present series, we designed individualised splints for each volunteer formed of 2 vinyl sheets, an internal sheet to which 6 discs were attached, and an external sheet that was fenestrated to permit contact of the vestibular surface of the discs with the saliva whilst protecting them from the action of the cheeks and tongue. Several discs were positioned on each hemi-arch and inserted towards the interdental area between 2 adjacent teeth in order to imitate an approximal PL-biofilm which is only minimally influenced by the shear forces of the oral soft tissues. This particular design ensured that the biofilm was not touched or disturbed during removal or repositioning of the appliance [34].

A number of solid substrates of different characteristics have been used in the published studies on PL-biofilm, including human enamel [17], [21], [22], [24], [45], bovine enamel [5], [25], [40], bovine dentine [27], [40], hydroxyapatite [23], and polished glass [2], [3], . Although the roughness of the surface of the substrate and its free energy are considered to be important factors for the *in vivo* growth of PL-biofilm [2], Netuschil et al. [21] found no major differences in the thickness of 2-day PL-biofilm on using enamel or glass disks; some authors recommend using glass to avoid any optical disturbance due to the known autofluorescence of enamel [21], [26]. On the basis of these findings, in the present series, glass disks were used for *in vivo* growth of the 2-day PL-biofilm.

In the majority of papers on PL-biofilm, the time for which the appliance remained in the oral cavity varied between 4 hours [24], [39], [44] and 7 days [4], [5], [42], depending on the type of PL-biofilm to be analysed. Specifically, Auschill et al. [2] demonstrated that the mean thickness of 48-hour PL-biofilm -with a range from 14 to 150 µm- was not affected by the position of the removable device within the oral cavity (maxillary buccal region *vs*. mandibular buccal region) or by the position of the disk (distal *vs*. mesial; right *vs*. left). In addition, Arweiler et al. [20] observed that the disk location in the oral cavity affected neither the mean viability values -with a range from 64% to 77%- nor the bacterial viability pattern in the 48-hour PL-biofilm. On the basis of these results, we designed individualised splints of the lower arch containing several disks, which were analysed from right to left; in a distal-mesial direction.

### Thickness, bacterial viability and structural characteristics of PL-biofilm

**Figure 5:**
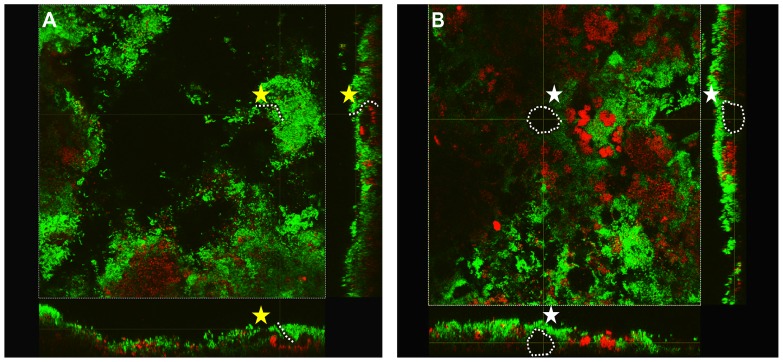
Visualization of channels (A, yellow star) and voids (B, white star) in a single cross sectional plane from X, Y and Z axis images obtained by the CLSM from a baseline sample. The presence of both channels and voids shows a heterogeneous architecture model.

In accordance with a high number of authors [2], [17], [20], [22], [27], in the present study, 2-day PL-biofilms analysed by CLSM showed an open complex, and heterogeneous architecture model with the presence of channels and voids and “bubble-like structures” (Figure 5).

The majority of authors who analysed the *in situ* PL-biofilm emphasised the great variation detected in PL-biofilm thickness between different individuals [2], [18], [22]; this condition was also observed in the present study (mean values of PL-biofilm thickness was 20 µm ranging from 11.75 µm to 33.00 µm), indicating that “*the height of the oral biofilms formed depended on the plaque-forming rate of the individual donors*” [22].

The *in situ* studies published on PL-biofilm viability over 2 and 3 day periods were relatively consistent, with authors reporting mean bacterial viability values between 60% and 77% [3], [20], [28]; accordingly, in the present series, the PL-biofilm viability was approximately 80%. Consequently, vital micro-organisms are located on and embedded in dead layers, which may be responsible for further plaque growth [21]. Dead bacteria may supplement living flora with all of the materials needed for rapid growth [21] and protect them against antibacterial influences in the oral environment [22]. For this reason, it has been stated that dead cellular material is a major component of the biomass during the initial stages of PL-biofilm accumulation and development [21], [22].

In some series, large inter-individual differences were found among the subjects with regard to their PL-biofilm viability distribution [27], so no general pattern for bacterial viability distribution could be described [27], [28]; accordingly, in the present study, the PL-biofilm viability ranged from 62% to 95%. However, it has been suggested that a relatively constant ecological environment exists in each volunteer, which obviously leads to a microbial identity pattern [20]. In this sense, Arweiler et al. [20] detected a great variation in the bacterial viability values in the 2-day PL-biofilms for the different biofilm layers, identifying 3 viability patterns: the first pattern was when a high number of dead bacteria were found in layers nearest the substrate, increasing in higher layers and then ending with low values at the outmost surface of the PL-biofilm; the second pattern was when these bacteria were superponed by new, vital bacteria, or some still vital or cultivable bacteria proliferated, forming a new layer of vital PL-biofilm; and the third pattern was when PL-biofilms started with high viability values adjacent to the substrate surface, and then decreased at their external aspect. In our study, despite the high variability detected in bacterial viability distribution, a viability pattern could be identified, which was based on a low viability percentage observed in layers nearest the substrate, increasing in higher layers.

In accordance with a large number of authors, 2-day PL-biofilm analysed *in situ* by CLSM showed an open complex, and heterogeneous architecture model and is characterised by the presence of a complex system of channels and voids described as an integral part of biofilm structure [2], [17], [20], [22], [27].

### 0.2% CHX: substantivity and influence on PL-biofilm thickness

To the best of the author's knowledge, there are few papers in which the CHX antimicrobial effect on PL-biofilm derived from a single application has been studied *in situ*, and the treatment was practiced *ex vivo* in both [27], [28]. In 2001, Zaura-Arite et al. [27] concluded that only minor and superficial bactericidal effects of 0.2% CHX were obtained on PL-biofilm, with a thickness less than 65 µm. On the other hand, it's very interesting to note that the subjects brushed their teeth twice a day (without the presence of intraoral appliances) with a NaF toothpaste [27], which could have conditioned the results obtained by these authors. On the contrary, von Ohle et al [28] demonstrated that CHX treatment significantly reduced the bacterial viability in the 3-day PL-biofilms during exposure to sucrose (67% in control biofilm compared to 2% and 0.7% in CHX-treated biofilms at 1 and 10 minutes, respectively). In accordance with these authors (28), in our study, the 0.2% CHX mouthrinse for 30 seconds significantly reduced the PL-biofilm viability (78% in basal conditions *vs* 5% at 30 seconds after the CHX application).

It has been stated that the concept “penetration” plays a more important role in *in situ* PL-biofilms, where a single application of an antimicrobial agent is tested [25]. In the present series, 0.2% CHX inactivated bacteria from the top down and layer by layer of PL-biofilm was killed with a similar efficacy in all regions. The rate and extent of antimicrobial agent penetration depend on factors including the biofilm structure and composition [4], [17], and perhaps, most importantly, biofilm thickness [46] as well as the physicochemical properties of the solute [4], [17]. On the other hand, although other very interesting aspects are based on solute penetration during brief exposure periods (<2 minutes), it is relatively unexplored [4]. In this sense, von Ohle et al. [28] used a simple diffusion model to calculate CHX concentration as a function of depth and time of application on a 3-day *in situ* PL-biofilm, assuming that a concentration of 0.1% CHX would be a clinically relevant concentration. The model predicts that if the thickness of the biofilm was reduced to 100 µm, the exposure time would be reduced to <2 minutes to achieve 0.1% CHX at the base of biofilm; if it was reduced to 30 µm, then it would only take 12 seconds. These observations could justify the results of 0.2% CHX antimicrobial activity on 2-day PL-biofilm obtained in the present study.

None of the previously described studies on CHX antimicrobial effect on PL-biofilm derived from a single application evaluated the CHX substantivity on PL-biofilm and the influence on its thickness [27], [28]. In the present series, the antimicrobial activity of 0.2% CHX was still detectable 7 hours after the mouthrinse, at which point the reduction in viability was 46%. A significant recovery in bacterial viability was detected in the post-mouthrinse biofilm samples collected after 3, 5 and 7 hours in comparison with the viability at 30 seconds after the CHX mouthrinse (especially in the biofilm layer 3 at 7 hours). With regard to the influence of 0.2% CHX mouthrinse on the PL-biofilm thickness, significant reductions in relation to baseline were detected at 1, 5 and 7 hours after application of antiseptic. In comparison to M-water, mean values of PL-biofilm thickness detected were significantly lower at 5 and 7 hours after M-0.2% CHX, which might suggest a possible anti-plaque effect derived from the single application of antiseptic.

### Substantivity of 0.2% CHX on saliva *vs*. PL-biofilm

Tomás et al. [11] stated that fluorescence assays could be particularly useful to simultaneously analyse the effect of antimicrobials that alter the cytoplasmic membrane integrity on different oral ecosystems. However, there are no studies published in the literature in which CHX substantivity on saliva and PL-biofilm was compared. Under basal conditions, PL-biofilm showed a significantly lower viability than that detected in salivary flora (a difference in the percentage of viable bacteria of 15%). The bacterial viability obtained in salivary flora agrees with previous results published by our research group [11], [33].

Numerous authors have demonstrated that bacteria growing in *in vitro* structured communities can be 10–1,000 times more resistant to antimicrobial treatment than those grown in planktonic phase [1], [47]. In the present series, bacteria growing in *in situ* 2-day PL-biofilm were 5 times more resistant (at 30 seconds) to 0.2% CHX mouthrinse than those present in salivary flora (viability percentages of 5% and 1%, respectively). However, 0.2% CHX mouthrinse showed higher substantivity (sustained antibacterial activity) on *de novo* PL-biofilm than on salivary flora at 5 and 7 hours after CHX application (viability percentage of 25% and 32% vs. 55% and 77%, respectively). This condition might be related to the slower growth rate of PL-biofilm [48], [49] or the presence of an open architecture with channels and voids, which would presumably provide direct communication between the oral environment and the enamel surface [17]. These “circulatory” channels and voids could have important implications for the movement of tooth damaging organic acids, bacterial toxins, and other antigens, as well as for the delivery of antimicrobial agents to the desired targets within the PL-biofilm [17]. In this sense, although it has been assumed that dead bacteria and exopolymeric substances impede fast penetration of the antimicrobials through the biofilm, on the contrary, other authors stated that PL-biofilm may also contribute to a reservoir function for antimicrobial agents [14].

A better understanding of the *in situ* antibacterial activity of CHX on different oral ecosystems could contribute to increase the clinical efficacy of CHX in the prevention and treatment of the oral biofilm-associated diseases.

## Conclusion

After a single mouthrinse of the 0.2% CHX formulation tested in the present study, the 2-day PL-biofilm presented a significantly higher resistance to this antiseptic *in situ* than that observed in salivary flora. However, this 0.2% CHX formulation showed a higher substantivity on PL-biofilm than on salivary flora at 5 and 7 hours after mouth-rinsing, which could be related to the slower growth rate of PL-biofilm and the possible reservoir function for antimicrobial agents associated with the undisturbed *de novo* PL-biofilm.
